# Screening and identification of dietary oils and unsaturated fatty acids in inhibiting inflammatory prostaglandin E_2_ signaling in fat stromal cells

**DOI:** 10.1186/1472-6882-12-143

**Published:** 2012-08-31

**Authors:** Diana Ruan, Shui-Ping So

**Affiliations:** 1 Science Department, Bellaire High School, Bellaire, TX, 77401, USA; 2 University of Houston, College of Pharmacy, Houston, TX, 77204, USA; 3 The Center for Experimental Therapeutics and Pharmacoinformatics, Department of Pharmacological and Pharmaceutical Sciences, College of Pharmacy, University of Houston, Houston, TX, 77204, USA

**Keywords:** Unsaturated fatty acid (UFA), Fish oil, Anti-inflammation, Prostaglandin E_2_ (PGE_2_), PGE_2_ receptor (EP), EP subtype-1 (EP_1_)

## Abstract

**Background:**

The molecular mechanisms of dietary oils (such as fish oil) and unsaturated fatty acids, which are widely used by the public for anti-inflammation and vascular protection, have not been settled yet. In this study, prostaglandin E_2_ (PGE_2_)-mediated calcium signaling was used to screen dietary oils and eight unsaturated fatty acids for identification of their anti-inflammatory mechanisms. Isolated fat/stromal cells expressing endogenous PGE_2_ receptors and an HEK293 cell line specifically expressing the recombinant human PGE_2_ receptor subtype-1 (EP_1_) were cultured and used in live cell calcium signaling assays. The different dietary oils and unsaturated fatty acids were used to affect cell signaling under the specific stimulation of a pathological amount of inflammatory PGE_2_.

**Results:**

It was identified that fish oil best inhibited the PGE_2_ signaling in the primary cultured stromal cells. Second, docosahexaenoic acid (DHA), found in abundance in fish oil, was identified as a key factor of inhibition of PGE_2_ signaling. Eicosapentaenoic acid (EPA), another major fatty acid found in fish oil and tested in this study was found to have small effect on EP_1_ signaling. The study suggested one of the four PGE_2_ subtype receptors, EP_1_ as the key target for the fish oil and DHA target. These findings were further confirmed by using the recombinant EP_1_ expressed in HEK293 cells as a target.

**Conclusion:**

This study demonstrated the new mechanism behind the positive effects of dietary fish oils in inhibiting inflammation originates from the rich concentration of DHA, which can directly inhibit the inflammatory EP_1_-mediated PGE_2_ receptor signaling, and that the inflammatory response stimulated by PGE_2_ in the fat stromal cells, which directly related to metabolic diseases, could be down regulated by fish oil and DHA. These findings also provided direct evidence to support the use of dietary oils and unsaturated fatty acids for protection against heart disease, pain, and cancer resulted from inflammatory PGE_2._

## Background

Fish oil and unsaturated fatty acids (UFAs) are widely used as supplements for prevention or treatment of inflammation, vascular diseases, pain, and cancer; however, the health benefits and molecular targets of these compounds have not been clearly identified yet. Nevertheless, many advances have recently been made by searching the molecular targets of fish oil. It is believed that the biological activity of fish oil is related to reducing the excess endogenous prostaglandin E_2_ (PGE_2_), which is synthesized through the inducible and inflammatory cyclooxygenase-2 (COX-2) pathway [1,2]. When cells undergo inflammatory stimulation, an endogenous fatty acid, arachidonic acid (AA), released from the cell membrane, is converted into an intermediator, prostaglandin H_2_, (by COX-2) and then further converted into PGE_2_ by PGE_2_ synthases [3-5]. There are three different PGE_2_ synthases in cells that have been identified, although the one most associated with inflammatory stimulation is the inducible microsomal PGE_2_ synthase-1 (mPGES-1) [6,7]. The inflammatory activity of PGE_2_ is mediated by its receptors (EPs) on the cell membrane. There are four subtypes of EPs, termed EP_1_, EP_2_, EP_3_ and EP_4_ which have been cloned, characterized [4], and identified in inflammatory tissues and cancers [8-13]. When the receptors receive excess PGE_2_, they immediately begin signaling and cause an increase in intracellular calcium (by EP_1_ and EP_3_) or cAMP (by EP_2_ and EP_4_) levels which leads to the biological functions of PGE_2_ – thus causing the inflammation that is directly related to the pathological processes of pain, vascular diseases, and cancer cell growth [4].

The COX-2/mPGES-1-pathway -induced excess PGE_2_ signaling plays a key role in inflammation and pain as confirmed by the non-selective nonsteroidal anti-inflammatory drugs (NSAIDs) like aspirin and selective COX-2 inhibitors (Vioxx and Celebrex), which specifically target COX activity [4,5]. From the century old drug, aspirin [5,14] to the modern COX-2 inhibitor, Celebrex [5], all NSAIDS have the same goal: inhibit COX’s ability to reduce excess PGE_2_ production and signaling. However, non-selective NSAIDs can cause severe stomach insult, such as bleeding.

In this study, we have screened several dietary oils and unsaturated fatty acids for identification of the particular oils and fatty acids with potential use for prevention and treatment of inflammation and its related diseases. We found that fish oil and DHA have the ability to inhibit the inflammatory PGE_2_ signaling receptor, EP_1_.

## Methods

### Materials

Medium for culturing the cell lines was purchased from Invitrogen. Other reagents were from Sigma. Dietary oils including olive, sesame, canola, fish oils were obtained from the Whole Foods brand 365. Unsaturated fatty acids (ALA, DHA, Erucic acid, EPA, oleic, LA, RA, and AA), Fluo-8 AM and lipase were obtained from Sigma (St. Louis, MO).

### Cell culture

The mouse stromal cells expressing endogenous subtype EPs were isolated from mouse fat tissue and cultured using Dulbecco's Modified Eagle's Medium (DMEM) medium with fetal bovine serum (FBS) as described previously [15]. The protocol for the animal fat cell extraction was approved by the University of Houston Institutional Animal Care and Use Committee.

### EP_1_ receptor pcDNA construction

A pAcSG-EP cDNA cloned by our laboratory was first subcloned into EcoRI/XhoI sites of pcDNA3.1 (+) expression vector to generate the plasmid of pcDNA: human EP_1_. The pcDNA vector has a Cytomegalovirus (CMV) promoter and geneticin (G418) as the selection antibiotic.

### Stable expression of recombinant human EP_1_ in HEK293 cells

The generation of the HEK293 cell line stably expressing human EP_1_ (HEK293-hEP_1_) has been described previously [16]. Briefly, HEK293 cells cultured in DMEM containing FBS (10%), antibiotics, and antimycotics were transfected with the purified pcDNA: human EP_1_ using the Lipofectamine 2000 method [16]. Approximately 48 hours after transfection, the cells were subcultured and incubated with G418 (selection antibiotic) for four weeks to generate the HEK293 cell line stably expressing human EP_1_ (HEK293-hEP_1_).

### Western blot analysis

#### Immunoblot analysis

The cultured cells were collected and washed with PBS. The proteins were separated by 7-10% (w/v) SDS-PAGE under denaturing conditions and then transferred to a nitrocellulose membrane. Bands recognized by individual primary antibodies were visualized with horseradish peroxidase-conjugated secondary antibody as described [16].

### Digestion of oils

One milliliter of each oil (olive, canola, sesame, and fish) was individually mixed with 10mg of lipase in 50 μl of PBS. The mixtures were vortexed and kept at 37°C for two hours and then centrifuged at 5,000 revolutions per minute (rpm) for five minutes. Supernatants were stored at 4°C.

### Determination of PGE_2_ signaling

The cultured cells were washed twice (1 mL medium/well), and then incubated with new cell culture medium (1 mL/well) containing calcium binding reagent, Fluo-8 (50 μg). After 30 minutes of incubation at 37°C, the cells were washed with serum-free medium three times to remove the excess Fluo-8 that did not enter the cells, and then observed under a Nikon fluorescence microscope (Nikon, Eclipse Ti) using a software (NIS Elements 2.35) designed specifically for calcium signaling. To start the signaling assay, PGE_2_ (50 μL, 0.12 μM) was added. The increasing fluorescence signal, generated by the calcium bound to Fluo-8 inside the cells through the binding of PGE_2_ to its receptor, was recorded under the fluorescence microscope and analyzed.

### Determination of the effects of the dietary oils on the PGE_2_-mediated receptor signaling in the cells

A procedure similar to that used above was performed for the determination of the effects of the dietary oils on PGE_2_-mediated receptor signaling. The only difference was that the PGE_2_ used was mixed with the digested individual oils at varied concentrations for 5 minutes before being added to the Fluo-8 loaded cells.

### Determination of the effects of individual fatty acids on the PGE_2_-mediated receptor signaling in the cells

Individual fatty acids dissolved in DMSO (10 μl, 10 μM)) were mixed with the PGE_2_ for five minutes, and then added into Fluo-8-loaded cells. The fluorescence changes were recorded under the fluorescence microscope. The PGE_2_ mixed with PBS was used as control.

### Data analysis

To observe the intensity of the fluorescence signaling mediated by the PGE_2_ in the absence and presence of the dietary oils and fatty acids, the specific effects of the dietary oils and fatty acids on the PGE_2_ signaling were identified by the comparison of the their signal intensities. After analyzing the control cells results, the results were plotted to show the difference between the oils and fatty acids. The experiments were repeated three times.

### Statistical analysis

Student’s *t*-test was used as the statistical analysis tool.

## Results

### The expression of endogenous PGE2 receptors in fat stromal cells

**Figure 1:**
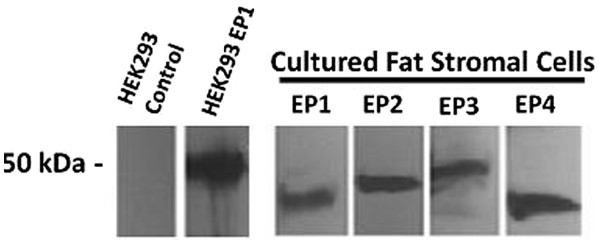
**Profiles of the Western blot analyses for PGE**_**2 **_**receptors (EPs).** 50 μg of the protein of the isolated cultured fat cells and HEK293 cells over expressing EP_1_ and control HEK293 cells were isolated by 12 % PAGE and then transferred to nitrocellulose membrane. The receptors were stained by specific subtype EP antibodies, as indicated.

During inflammation, COX-2 and mPGES-1 genes in the cells are upregulated, which increase the enzyme levels to metabolize the endogenous unsaturated fatty acid, arachidonic acid (AA). AA is converted into a mediator (PGH_2_) by COX-2, and then further converted into PGE_2_ by PGES enzymes. This pathway can result in excess PGE_2_, which binds to receptors on the surface of cells. The binding causes the receptor to couple to internal proteins and trigger an increase in secondary messenger molecules, such as calcium and cAMP inside the cell. This signaling has been identified as a pathogenic factor promoting cancer, heart diseases, and arthritis/pain. The fat stromal cells were isolated from the mouse fat tissue and cultured. The PGE_2_ receptors were identified by Western blot analysis using EP subtype-specific antibodies (Figure 1). Among the four EPs, EP_1_and EP_3_ mediates calcium mobilization, but EP_2_ and EP_4_ mediate cAMP changes. For determination of the live cell signaling, EP_1_ is the best choice since the calcium mobilization can be monitored while the cells are still alive (in culture).

### Mimicking the gastrointestinal tract to digest oils

**Figure 2:**
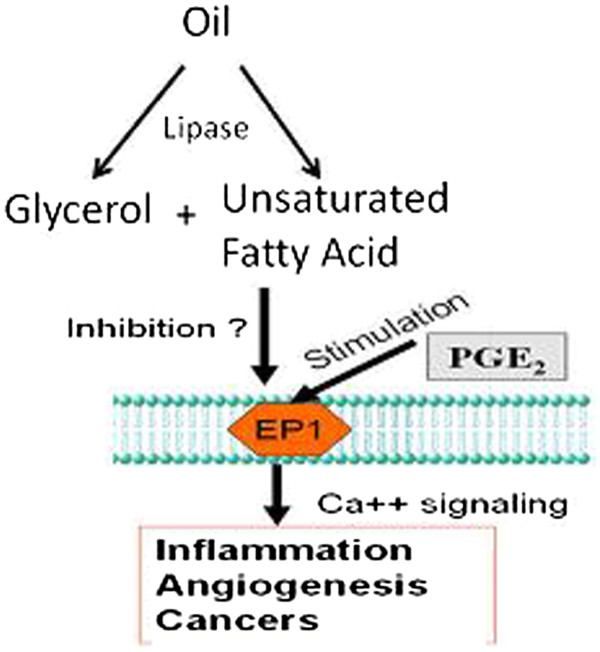
**Schematic representation showing the breakdown of digested oils with lipase.** The fatty acid component of the dietary oil is absorbed into the body through the cell membrane as shown and then lead to several actions/diseases through the designated receptor, as indicated.

The dietary oils taken into the gastrointestinal tract must be digested into fatty acids and glycerol, and then absorbed into the body. To mimic this step, the oils were incubated with lipase (produced by the pancreas) for 120 minutes at 37°C. The lipase was able to break down the oils into fatty acid and glycerol, as shown in Figure 2.

### Identification of the excessive inflammatory PGE_2_ induced EP_1_ signaling in the fat stromal cells using the highly sensitive Fluo-8 as a calcium indicator

**Figure 3:**
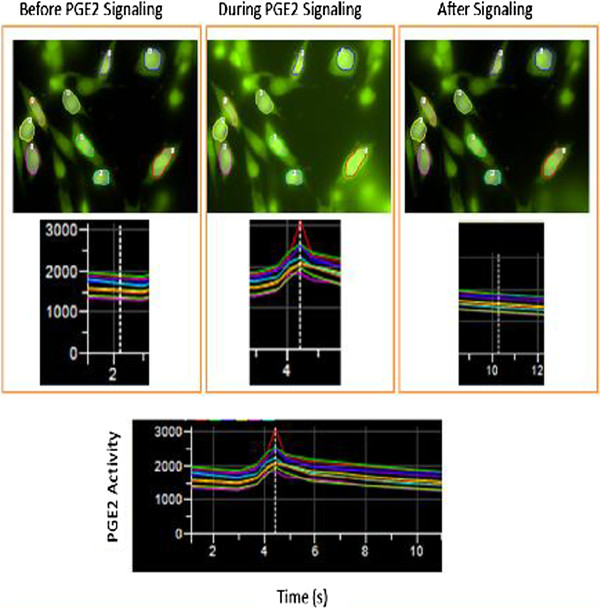
**Photographs and corresponding fluorescence intensity graphs for individual fat stromal cells before, during, and after PGE**_**2 **_**signaling.** Each stromal cell was circled on the screen using a different color to measure their intensity level and the PGE_2_-induced signaling was recorded using the corresponding matching colors. The graphs directly below the photographs represent the cell signaling before, during, and after the addition of PGE_2_ (0.12 μM). The graph on the bottom is the complete signaling pattern for all cells.

The micromolar concentration of PGE_2_ which can cause inflammation was added to the cells to see the response through EP_1_ calcium signaling. However, the traditional Fura-2 calcium assay for live cells is not sensitive enough and therefore not very promising for determination of endogenous EP_1_ activity. Thus, Fluo-8, one of the newer calcium indicators was used based on its ability to increase fluorescence signals by 100–250 times when the compound forms a complex with calcium. Based on this principle, the membrane permeable Fluo-8 was introduced to the cultured fat stromal cells expressing endogenous EPs. The fluorescent signal increase after the addition of exogenous PGE_2_ represents the increase in intracellular levels of calcium from the inflammatory PGE_2_ bound to its receptor (Figure 3). During the assay, the fluorescence intensity of the live cells was monitored using the designated fluorescence microscope. The background signal of the Fluo-8 in the cells is shown in Figure 3 (before PGE_2_ addition). After the PGE_2_ was added and signaling started, the fluorescence intensity of the cells increased due to the presence of calcium signaling induced by the PGE_2_ bound to its receptors in the cells. The cell signaling only lasts a few seconds and then returns to the base line. Figure 3 shows the individual cells’ response to the calcium signaling after binding to PGE_2_.

### Determination of the effects of the digested dietary oils on PGE_2_-mediated receptor signaling in the live cells

**Figure 4:**
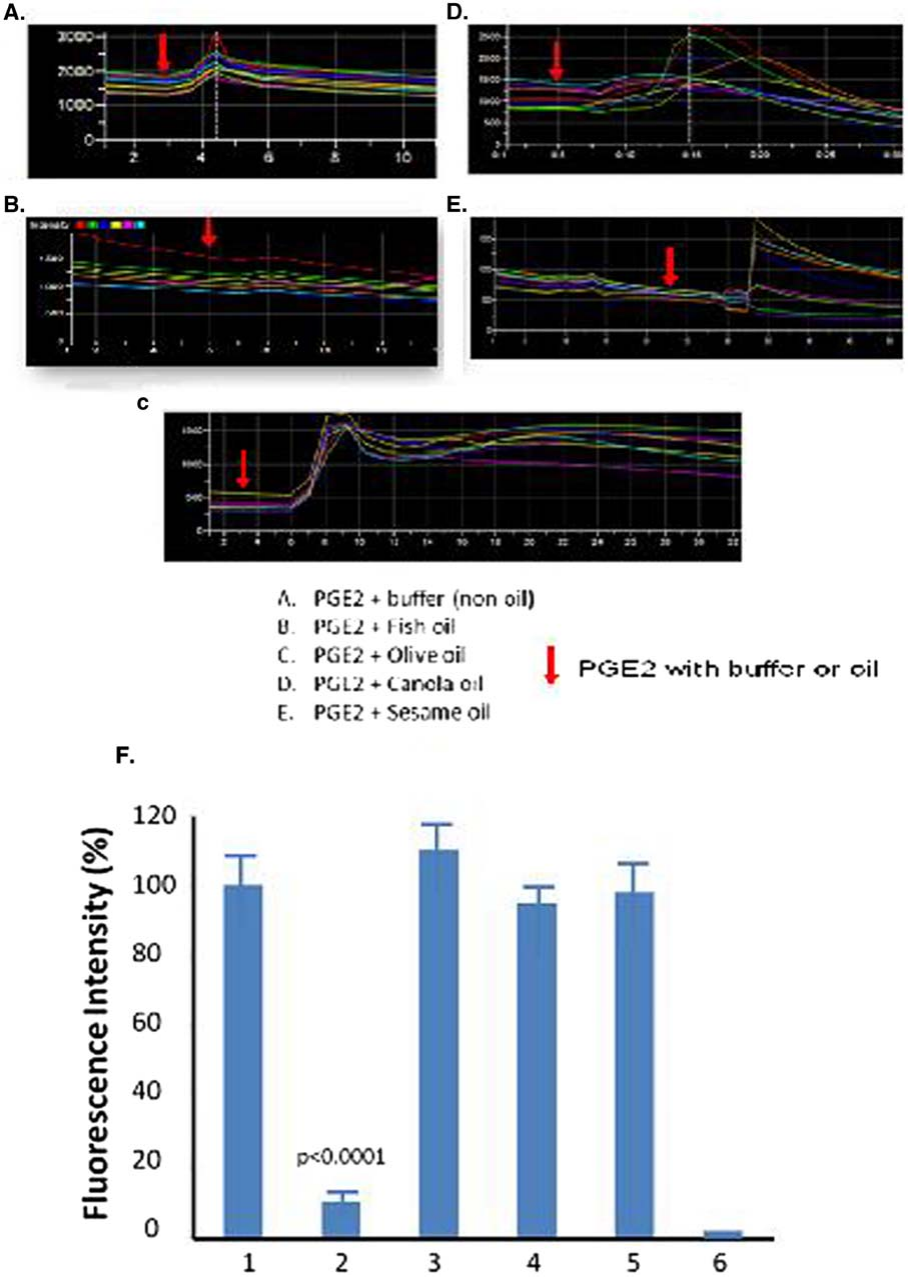
**Profiles of the inhibition of the oils on PGE**_**2 **_**signaling.** Following the same procedures as those mentioned in Fig. 3, the PGE_2_ signaling was recorded for stromal cells in the presence of PGE_2_ with buffer (**A**.), fish oil (**B**.), olive oil (**C**.), canola oil (**C**.), or sesame oil (**E**.). The arrow indicates the addition of PGE_2_ plus buffer/oil. Comparison of the inhibition of the oils on PGE_2_ signaling (n = 4) was showed in **F** (1, buffer; 2, fish oil; 3, olive oil; 4, canola oil; 5, sesame oil, and 6, HEK293 control).

The PGE_2_ (0.12 μM) was mixed with ~1.0 μl of four individual, digested dietary oils including fish, olive, canola and sesame oils, and then was added into the cultured cells. The profiles of the PGE_2_-mediated calcium signaling (in the presence of the oils) were recorded and shown in Figure 4. The data revealed that the only oil which strongly inhibited PGE_2_ signaling was fish oil (Figure 4B, F). Other oils showed interference of the signaling through changes in signaling patterns, but no significant inhibition (Figure 4C-E). The significant inhibition of the fish oil on the PGE_2_ signaling was further compared and plotted in Figure 4F.

### PGE_2_ signaling affected by the different concentrations of fish oil

**Figure 5:**
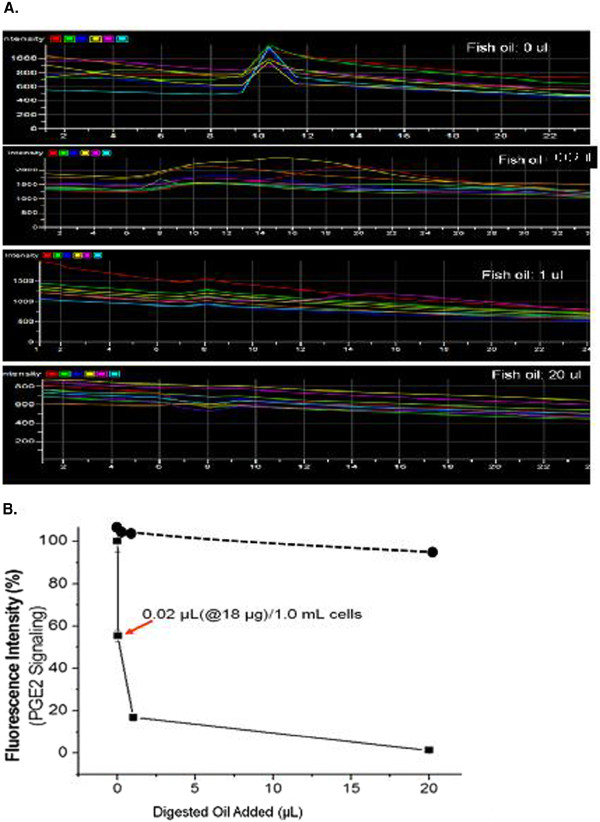
**A) Profiles for increasing PGE**_**2 **_**signaling inhibition.** Increasing amounts of fish oil (as labeled) were added to the stromal cells and the signaling was recorded. **B**). Dose response curve for increasing PGE_2_ signaling inhibition in the stromal cells with fish oil. The IC50 determination by converting Figure 4A inhibition peaks into curve and using approximately 0.02 ul of the fish oil was indicated.

To further confirm that fish oil can inhibit PGE_2_ signaling, increasing concentrations of the digested fish oil were added to the PGE_2_ cell signaling system. Figure 5A showed the entire profiles of PGE_2_ signaling decreasing with the increasing digested fish oil concentrations. The data were further calculated for the peak values, and then plotted in Figure 5B, which provided a dose response curve for identification of 50% inhibition (IC50) of the oil concentration. The results revealed that 0.02 μl of the fish oil was able to give approximately 50% inhibition for the PGE_2_ signaling (Figure 5B). Based on the estimation that UFAs in the fish oil is approximately 80%, then 0.02 μl of digested fish oil contained approximately 50% UFAs. Thus, 0.02 μl (18 μg) oil is equivalent to 9 μg of the mixed UFAs in one mL assay, which gives approximately a 27 μM concentration based on average MW 300 for the UFAs.

### The effects of individual UFAs on the PGE_2_-mediated receptor signaling in the cells

**Figure 6:**
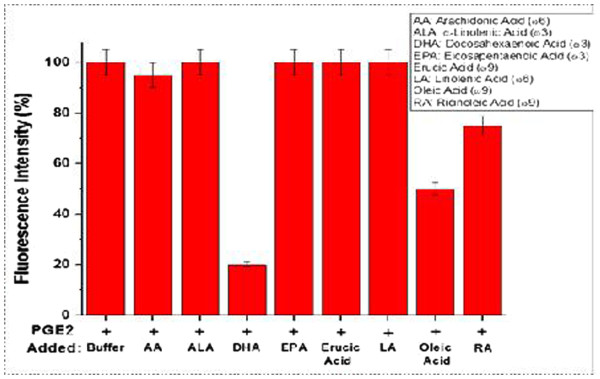
**Comparison of the effects of the UFAs on PGE**_**2 **_**signaling through the binding to the EP**_**1 **_**receptor.** The intensity is plotted as percentage (n = 3). The P values of DHA and EPA compared to others are <0.01 and <0.05, respectively.

The dietary oils are formed by different unsaturated fatty acids and glycerol. In order to find out the reasons why fish oil inhibits PGE_2_ signaling, eight unsaturated fatty acids were tested for their inhibition of PGE_2_ signaling using the same calcium assay. DHA had the strongest inhibition of PGE_2_ signaling (Figure 6). EPA and other fatty acids did not have significant inhibition at those concentrations and oleic acid showed 50% inhibition. Fish oils contain large amounts of DHA and EPA.

### Confirmation of the fish oil specifically inhibiting EP_1_ calcium signaling using recombinant human EP_1_ expressed in HEK293 cells

**Figure 7:**
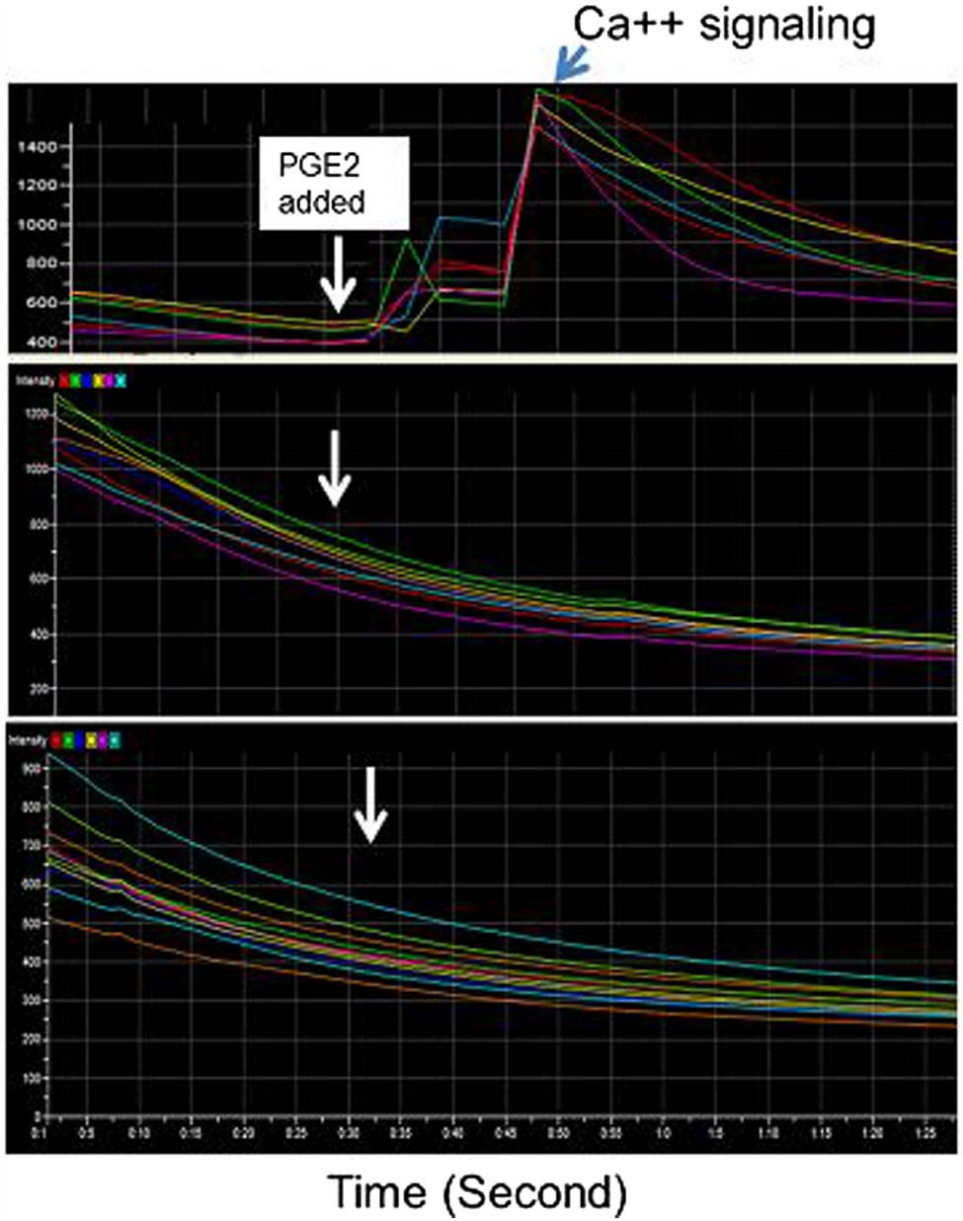
**Profiles for increasing PGE**_**2 **_**signaling inhibition.** Increasing amounts of fish oil were added to the HEK293-EP_1_ cells following the same procedures described in Figure 5 and the signaling was recorded. The HEK293 cell stably expressing recombinant human EP_1_ receptor was cultured and used as target to replace the fat stromal cells shown in Figure 5.

To further confirm whether the results obtained from mouse fat stromal cells could be applied to human use, a recombinant human EP_1_ was over expressed in the human cell line (HEK293) which has minimal expression of the other subtype EPs, and then used as a target for the fish oil inhibition assay similar to that described in Figure 5. Similar results were observed, in which the digested fish oil has an identical pattern for inhibiting the human EP_1_ signaling (Figure 7), which is similar to that of the fat stromal cells (Figure 5).

## Discussion

COX-2 inhibitors can increase the risk for heart disease, which is a serious side effect because the COX-produced intermediator, PGH_2_, is also a substance used for producing the vascular protective molecule, prostacyclin [5]. Thus, finding supplements as alternatives to the selective and non-selective NSAIDs to reduce and prevent inflammation is of high importance. Some UFA-rich dietary oils with unsaturated fatty acids such as fish oil have been found to have anti-inflammatory functions. Most reports focused on the observation and confirmation that dietary oils could reduce PGE_2_ biosynthesis through competition with AA on the substrate-binding site of COXs [17,18]. However, we found that the fish oil was not very effective in inhibiting COX-2/mPGES-1 activities via inhibition of the inflammatory PGE_2_ biosynthesis, and thus may be doing so through another mechanism. Based on the fact that PGE_2_ is derived from a UFA, and that UFA molecules have similar structures to PGE_2_, we can safely say that molecules which have similar structures to PGE_2_ may bind to some target molecules, such as EPs, and in turn block PGE_2_ signaling. In this case, the similar UFA molecules may act as antagonists against inflammation. Many drugs have been developed for treatment of diseases based on the antagonist principle. The search for an “antagonist” to compete with PGE_2_ in binding to the receptor and block its signaling is an important step towards finding something that can reduce PGE_2,_ yet maintain COX activity for production of the vascular protector, prostacyclin. This information has led us to hypothesize that if dietary oils contain unsaturated fatty acids with similar structures to that of PGE_2_, then fish oil and certain omega-3 fatty acids may have the ability to block PGE_2_ from binding to its receptors and then inhibit signaling, resulting in reduced inflammation. In this paper, we tested this hypothesis and found a novel mechanism of dietary oils and UFAs showing anti-inflammatory potential through inhibiting EP signaling. The study has provided important evidence that fish oil and one of its major ingredients, DHA, have the ability to inhibit PGE_2_ signaling, which highlights the benefit of taking fish oil as a supplement for health, including preventing inflammation, cancers and heart diseases.

Many studies have shown that the use of UFAs, such as omega-3 fat acid and the UFA-rich dietary oils, can benefit in the prevention and reduction of inflammation that is involved in promoting heart disease, cancer, arthritis, and pain. However, little information is available regarding the effects of the dietary oils and UFAs on specific inflammatory PGE_2_ signaling (mediated by receptors) that are considered better targets for developing novel therapeutic interventions against inflammation. Our study has found that fish oil has a significant effect on inhibition of PGE_2_ binding to the EP_1_ receptor and thus inhibiting the signaling (Figure 5). The observation was further confirmed by a dose response assay, in which the IC50 was established (Figure 6). These results suggested that the fish oil could also likely bind to the other subtype PGE_2_ receptors, EP_2_, EP _3_ and EP _4_ and inhibit their signaling. This led to the conclusion that dietary fish oil can reduce PGE_2_-mediated inflammation. Therefore, consuming fish oil can reduce major risk factors of cancers, heart disease, arthritis, and pain. In contrast, olive oil, canola oil and sesame oil have altered PGE_2_ signaling patterns (delaying or prolonging signaling, Figure 5), but have less effects on inhibition of PGE_2_-mediated calcium signaling. Thus, taking these oils may not yield results as strong as fish oil for the prevention of cancers, heart disease, arthritis, and pain caused by excess inflammatory PGE_2_.

To further identify the active ingredient in the fish oil involved in the inhibition of PGE_2_ binding to its receptors, screenings of the inhibitory activities of the UFAs (major ingredients in the dietary oils) were performed. Among the eight UFAs present in the dietary oils, DHA best inhibits PGE_2_-signaling. Data analysis has determined that fish oil contains high concentrations of DHA compounds, which provides strong scientific evidence supporting the study in which fish oil has superior inflammation inhibiting abilities. Previously, researchers had found that fish oil high in DHA and low in EPA reduced inflammation, but were unaware of the mechanisms behind this conclusion [17,18]. This study has provided a novel finding and evidence that fish oil and DHA have anti-inflammatory effects through inhibiting PGE_2_ receptor signaling.

The study not only provided the mechanism behind fish oil and DHA acting on receptor levels, but also quantitatively measured the effect of the fish oil on inhibiting PGE_2_ signaling, which provides important information on how to effectively supplement with fish oil. In Figure 6B, the IC50 for fish oil is about 0.02 μl (0.018 mg) in a total of 1000 μl (1.0 ml) cell solution. Thus, the IC50 for fish oil is approximately 18 mg/L or 54 μM (using the average molecular weight of fatty acids with 300 Daltons). There are 4–5 liters of bloods in a 150 pound body. This means that consuming 100 mg fish oil should yield IC50 results. If given 5 to 10 times the IC50, then taking 500 –1000 mg fish oil daily is recommended based on the findings in this study.

## Conclusion

Finally, we conclude that fish oil is a promising dietary oil used to prevent and reduce inflammation-mediated diseases, such as heart diseases, cancers, arthritis, and pain. The mechanism behind the benefits of fish oil is not only to interfere with PGE_2_ biosynthesis as reported previously, but more importantly the fish oil has the ability to inhibit the inflammatory factor, PGE_2_ binding to its receptor and thereby reduce the inflammatory signaling. In other words, fish oil can be taken by people striving to reduce risks of heart disease, cancer, and arthritis. This study also revealed that the mechanism behind the inhibiting of PGE_2_ signaling of fish oils comes from the rich concentrations of DHA, which have the ability to directly inhibit PGE_2_ signaling. These findings may greatly affect people that consume dietary oils on a daily basis, and also those concerned with protecting themselves against health risks, cancer and pain. This study was an attempt to find a novel mechanism for the fish oil and UFA on inhibition of the inflammation. However, it should be noted that other possibilities and pathways should not be excluded.

## Competing interests

The authors declare that they have no competing interests.

## Authors’ contributions

DR carried out the cell culturing and calcium signaling assay, participated in the Western blot analysis, and prepared the Figures and drafted the manuscript. SS carried out the cell line construction and Western blot, participated the cell signaling assay and finalized the manuscript preparation. Both authors read and approved the final manuscript.

## Pre-publication history

The pre-publication history for this paper can be accessed here:

http://www.biomedcentral.com/1472-6882/12/143/prepub
